# Outcomes of a novel algorithm for levator muscle plication surgery in congenital blepharoptosis

**DOI:** 10.1186/s12886-024-03287-y

**Published:** 2024-01-16

**Authors:** Ahmed N Kotb, Moustafa A. Salamah, Ahmad S. Khalil, Reem Amir Kamal Dessouky

**Affiliations:** Department of Ophthalmology, Faculty of Medicine, Zagazig University, Zagazig, Egypt

**Keywords:** Nomogram, Levator plication, Congenital ptosis

## Abstract

**Aim:**

to assess the outcomes of a novel algorithm for the calculation of the amount levator muscle plication in congenital blepharoptosis surgery.

**Methods:**

this retrospective comparative study included 34 patients with congenital ptosis subjected to levator muscle plication surgery during the period from October 2021 to November 2022. They were divided into two groups. Group A: the amount of levator muscle plication was calculated by a traditional formula [(amount of ptosis x 3) + 9 mm in cases with good levator function or (amount of ptosis x 3) + 11 mm in cases with fair levator function]. Group B: the amount of levator muscle plication was calculated by a novel nomogram [the result of the traditional formula was modified by subtracting 4 mm if the calculated amount was ≥ 15 mm or subtracting 3 mm if the calculated amount was < 15 mm]. Demographic data, baseline ptosis characteristics and postoperative results at 1st week, 1st month, 3rd month and 6th month were compared between the groups. Primary outcome measure was postoperative Marginal Reflex Distance (MRD1). Secondary outcome measures were lid contour, lid crease and any reported complications.

**Results:**

Group A included 20 eyes of 18 patients while Group B included 20 eyes of 16 patients. The mean amount of levator muscle plication was 16.98 ± 2.44 mm and 13.48 ± 2.42 mm in group A and group B respectively. The difference between the two groups was highly statistically significant (*p* < 0.001). Mean MRD1 at the 1st postoperative week was 4.95 ± 0.37 mm in group A and 4.08 ± 0.64 mm in group B. This difference was highly statistically significant (*P* < 0.001). Overcorrection was seen in 8 (40%) eyes in group A and 1 (5%) eye in group B. The difference was statistically significant between the two groups (*p* = 0.008). Undercorrection was seen in only 1 (5%) eye in group B. No other complications were reported. Surgical success was achieved in 12 (60%) eyes in group A versus 18 (90%) eyes in group B. The difference between the two groups was statistically significant (*p* = 0.03).

**Conclusion:**

our novel nomogram for the calculation of the amount levator muscle plication in congenital blepharoptosis surgery is effective in achieving a satisfactory postoperative MRD1.

## Introduction

Congenital ptosis is a common oculoplastic condition characterized by a lower-than-normal position of the upper eyelid dating from birth or shortly afterwards [1]. Treatment is mainly surgical, and the choice of the appropriate surgical technique depends mainly on the degree of levator function. When the levator function is equal to or greater than 5 mm, levator muscle resection or plication is preferred [2]. The levator resection technique is considered the gold standard as it is a well-known procedure with predictable results. In recent years, levator plication has become more widely adopted in cases of congenital ptosis due to its simplicity [3].

Various methods are available to calculate the amount of levator resection based on levator function, MRD1 and intraoperative lagophthalmos [4]. However, to the best of our knowledge, there is no formula specifically tailored for the levator muscle plication technique. The correct amount of plication is based mainly on the expertise of the skilled surgeon. From the results of our previous cases that underwent levator plication, we developed an algorithm especially for this technique. The aim of this study was to compare the outcomes of our novel algorithm and a traditional formula we used to calculate the amount of levator muscle plication for congenital blepharoptosis surgery.

## Patients and methods

This is a retrospective nonrandomized comparative study that was conducted in compliance with the Declaration of Helsinki and was approved by the Institutional Review Board of the Faculty of Medicine, Zagazig University, Egypt (ZU-IRB#:9941-12-10-2022). The medical records of patients that were diagnosed with congenital ptosis and subjected to levator muscle plication surgery during the period from October 2021 to November 2022 were retrieved. Patients with mild to moderate simple congenital ptosis and good to fair levator function were included in the study. Cases with severe congenital ptosis, poor levator action, recurrent or acquired ptosis, or negative Bell’s phenomenon and guardians refusing to participate in the study were excluded. All patients were subjected to thorough history taking and complete ophthalmic examination with special attention to MRD1, levator action, lid crease and Bell’s phenomenon. Ptosis was classified according to the amount of lid drooping into mild (< 2 mm), moderate (2–4 mm), and severe (> 4 mm). Levator function was graded according to the eyelid excursion as excellent (≥ 13 mm), good (8–12 mm), fair (5–7 mm), and poor (≤ 4 mm).

Based on the method of calculation of the amount of levator muscle plication, patients were divided into two groups. Group A in which the intended amount of levator muscle plication was calculated by the traditional formula. Group B in which the intended amount of levator muscle plication was calculated by our novel nomogram.

The amount of levator muscle plication was calculated for each eye preoperatively based on the degree of ptosis and amount of levator action.

Traditional formula (Group A): In cases with good levator function: amount of plication = (amount of ptosis x 3) + 9 mm. In cases with fair levator function: amount of plication = (amount of ptosis x 3) + 11 mm.

Novel algorithm (Group B): After calculating the amount of levator plication using the traditional formula, the result was modified as follows: If the calculated amount to be plicated was ≥ 15 mm, we subtracted 4 mm and performed the plication using the new amount calculated. If the calculated amount to be plicated was < 15 mm, we subtracted 3 mm and performed the plication using the new amount calculated.

### Surgical technique

All surgeries were performed under general anesthesia by a single surgeon. The surgical site and eye lashes were cleaned with Povidone iodine. The upper eyelid skin crease was marked, then using a no. 15 Beard-Barker blade, the skin was incised along the whole mark length, then the orbicularis muscle was cut and separated with scissors. Dissection was done in the submuscular plane downwards to expose the anterior tarsal surface and upwards to expose the orbital septum. The septum was then opened allowing the preaponeurotic fat pads to bulge. A small portion of the fat pads was clamped, cut, and cauterized to allow good exposure of the underlying levator muscle and better intraoperative visualization. The levator muscle was plicated using double armed 5/0 Ethibond suture. The first suture was taken in the center at the level of the pupil. One needle was passed horizontally through partial thickness of the tarsus about 2 mm below the superior tarsal border. The other needle was passed horizontally through partial thickness of the levator aponeurosis at a level that achieved the calculated amount of plication. Another two sutures were taken medial and lateral to the first one in a similar fashion to adjust the lid contour. Finally, the skin was closed using 6/0 vicryl suture; three interrupted skin crease forming sutures were taken, followed by simple skin to skin sutures in between along the whole length of the wound. At the end of surgery, an antibiotic-steroid eye ointment was applied on the wound and an eye bandage was placed.

### Postoperative care and follow up

All patients were instructed to apply antibiotic eyedrops four times daily, an antibiotic ointment at night, and a lubricant every 2 h. These medications were gradually tapered over a period of one month. Follow up visits were scheduled on the 1st week, 1st month, 3rd month and 6th month postoperative. MRD1, lid contour, lid crease and any complications were recorded and photographed in each visit to document the postoperative results.

Primary outcome measure was postoperative MRD1. Satisfactory correction was defined as MRD1 = 3–5 mm. Undercorrection was defined as MRD1 < 3 mm and overcorrection as MRD1 > 5 mm. Surgical success was defined as MRD1 of 3–5 mm and eyelid height asymmetry < 0.5 mm. Secondary outcome measures included lid contour, lid crease and any reported complications.

### Statistical analysis

All data were collected, tabulated, and statistically analyzed using SPSS version 25. Quantitative variables were expressed as the mean ± SD. Student’s t-test was used to compare quantitative continuous data between the two groups. A repeated measures (ANOVA) was used to assess quantitative data at different measurement times in the same group. Categorical data were expressed as frequency. Chi-squared or Fisher exact test was used to compare categorical data between the two groups. A p-value < 0.05 was considered statistically significant.

## Results

This study included 40 eyes of 34 patients with simple congenital ptosis that underwent transcutaneous levator muscle plication surgery. Group A included 18 patients, 10 males and 8 females, with a male to female ratio of 1.25:1. Group B included 16 patients, 9 males and 7 females with a male to female ratio of 1.29:1. The mean age of patients was 8.22 ± 2.26 years and 8.75 ± 2.21 years in group A and group B respectively. The difference between the two groups was not statistically significant regarding patients’ demographic data (*p* > 0.05). 16 (88.9%) patients had unilateral ptosis and 2 (11.1%) patients had bilateral ptosis in group A, while 12 (75%) patients had unilateral ptosis and 4 (25%) patients had bilateral ptosis in group B (Table 1).

**Table 1:** Baseline patient characteristics in the studied population

	Group A (18 patients)		Group B (16 patients)		P value^†^
Age (years) Mean ± SD Range	8.22 ± 2.26 5–13		8.75 ± 2.21 5–18		0.37^*^
Sex Male Female	N	%	N	%	0.97^**^
	10 8	55.6 44.4	9 7	56.2 43.8	
Laterality	N	%	N	%	
Bilateral Unilateral	2 16	11.1 88.9	4 12	25 75	0.39^**^

^†^*P-*value > 0.05 is not significant, *t-test **Chi-squared/Fisher exact test

Levator function was good in 10 (50%) eyes and fair in 10 (50%) eyes in group A versus good in 9 (45%) eyes and fair in 11 (55%) eyes in group B. Mean amount of ptosis in group A was 2.33 ± 0.59 mm and in group B was 2.48 ± 0.64 mm. Mean preoperative MRD1 was 1.68 ± 0.59 mm and 1.53 ± 0.64 mm in group A and group B respectively. There was no statistically significant difference between the two groups as regards preoperative ptosis characteristics (*p* > 0.05). The mean amount of levator muscle plication in group A was 16.98 ± 2.44 mm and in group B was 13.48 ± 2.42 mm. The difference between the two groups was highly statistically significant (*p* < 0.001) (Table 2).

**Table 2:** Preoperative ptosis characteristics and calculated amount of levator muscle plication in both study groups

	Group A (20 eyes)		Group B (20 eyes)		P value
Levator function	N	%	N	%	
Good Fair	10 10	50 50	9 11	45 55	0.75^**†^
Ptosis (mm) Mean ± SD Range	2.33 ± 0.59 1–3		2.48 ± 0.64 1-3.5		0.45^*†^
Preoperative MRD1 (mm) Mean ± SD Range	1.68 ± 0.59 1–3		1.53 ± 0.64 0.5–3		0.45^*†^
Amount of plication (mm) Mean ± SD Range	16.98 ± 2.44 12–20		13.48 ± 2.42 9-17.5		< 0.001^*††^

^†^*P-*value > 0.05 is not significant, ^††^*P*-value < 0.001 is highly significant, ^*^t-test ^**^Chi-squared test

**Fig. 1:**
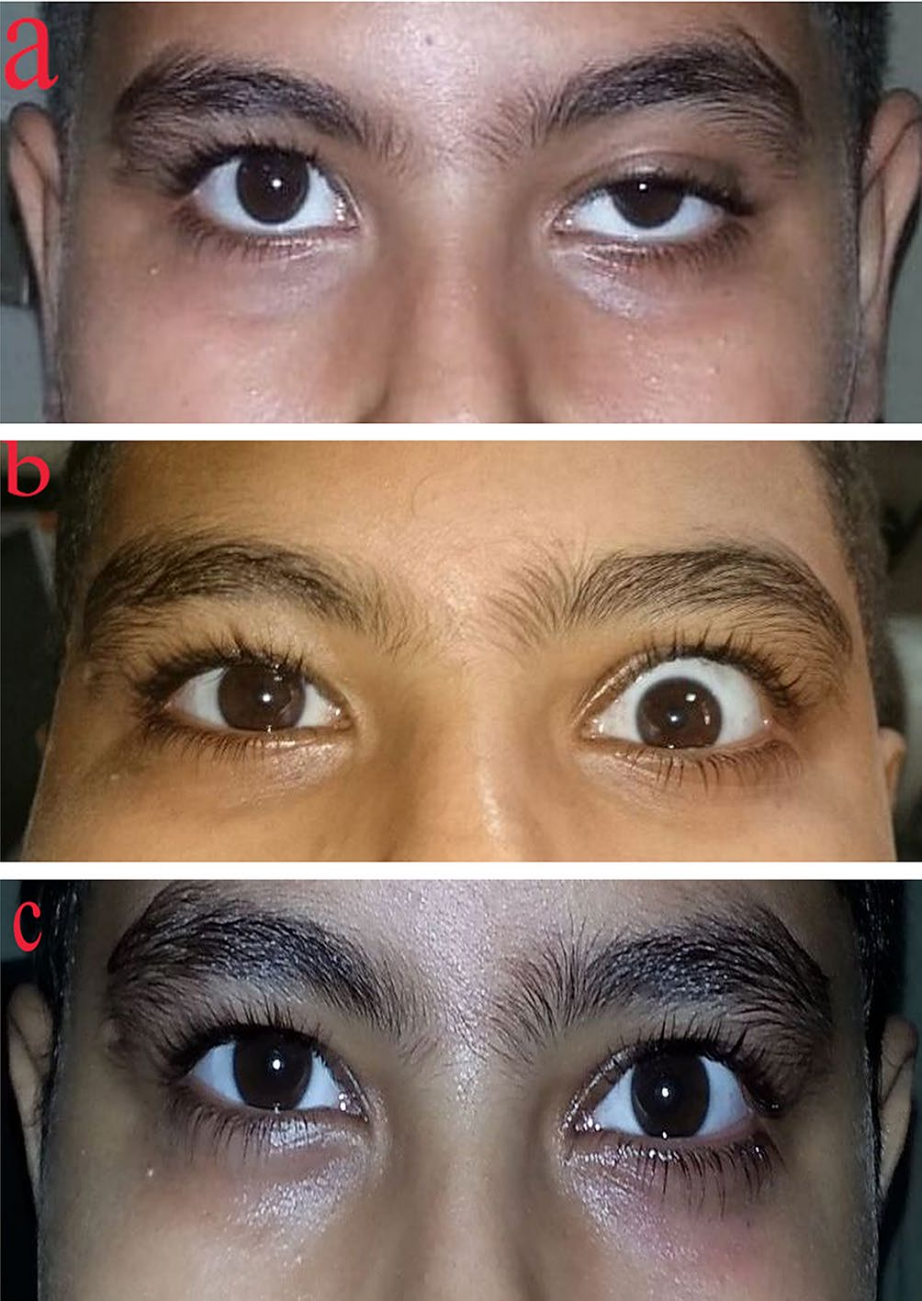
photo of patient in group A, (**a**) preoperative ptosis in left eye, (**b**) postoperative overcorrection, and (**c**) normal eyelid position following reoperation

Mean postoperative MRD1 at the 1st postoperative week was 4.95 ± 0.37 mm in group A and 4.08 ± 0.64 mm in group B. The difference between the two groups was highly statistically significant (*P* < 0.001) (Table 3). 8 (40%) eyes in group A (Figs. 1) and 1 (5%) eye in group B showed overcorrection at the 1st week. There was a statistically significant difference between the two groups regarding the incidence of overcorrection (*p* = 0.008) (Table 4). Immediate lid traction was performed to all eyes and patients were advised to apply a lubricant gel four times daily in addition to the usual postoperative regimen. More frequent follow up visits were scheduled to assess the lid level and examine the cornea to exclude exposure. Overcorrection was adjusted by lid traction in 4 (20%) eyes in group A and 1 (5%) eye in group B. 4 (20%) eyes in group A did not respond to lid traction and required reoperation at the 2nd postoperative week. The plication sutures were removed, and new sutures were taken to plicate less amount of levator muscle (levator muscle recession). No cases developed corneal exposure due to overcorrection.

Under correction was seen in only 1 (5%) eye in group B. No eyes in group A showed under correction. This difference was not statistically significant (*p* > 0.05) (Table 4). The case of under correction underwent a levator muscle resection surgery at the 1st postoperative month that was successful.

Mean postoperative MRD1 at 1st month, 3rd month, and 6th month postoperative in group A was 4.45 ± 0.72 mm, 4.28 ± 0.55 mm, and 4.28 ± 0.55 mm respectively and in group B was 4.28 ± 0.81 mm, 4.13 ± 0.61 mm, and 4.13 ± 0.61 mm respectively. The difference in mean postoperative MRD1 was not statistically significant between the two groups at the 1st, 3rd and 6th months (*p* > 0.05). This was attributed to correction of overcorrected cases in group A by the end of the second postoperative week. Both groups showed a highly statistically significant improvement in preoperative MRD1 compared with postoperative MRD1 at all follow up visits (*p* < 0.001). MRD1 stabilized by the 3rd postoperative month in both groups, and we didn’t report any cases of recurrence till the last follow up visit (Table 3).

**Table 3:** Changes in MRD1 among both studied groups

	Group A	Group B	P value^*^
Preoperative Mean ± SD Range	1.68 ± 0.59 1–3	1.53 ± 0.64 0.5–3	0.45^†^
After 1 week Mean ± SD Range	4.95 ± 0.37 4–6	4.08 ± 0.64 3–5.5	< 0.001^††^
After 1 month Mean ± SD Range	4.45 ± 0.72 3.5–6	4.28 ± 0.81 3–6	0.52^†^
After 3 months Mean ± SD Range	4.28 ± 0.55 3.5–5	4.13 ± 0.61 3–5	0.15^†^
After 6 months Mean ± SD Range	4.28 ± 0.55 3.5–5	4.13 ± 0.61 3–5	0.15^†^
P value within group^**^	< 0.001^††^	< 0.001^††^	

^†^*P-v*alue > 0.05 is not significant, ^††^*P-v*alue < 0.001 is highly significant, ^*^t-test ^**^Repeated measures ANOVA

Surgical success was achieved in 12 (60%) eyes in group A versus 18 (90%) eyes in group B. The difference between the two groups was statistically significant (*p* = 0.03) (Table 4). All patients had a satisfactory cosmetic appearance. We did not report any cases of lid contour, lid crease, or sulcus abnormalities. No other postoperative complications occurred throughout the study period.

**Table 4:** Postoperative outcomes among both studied groups

	Group A	Group B	P value^*^
	N (%)	N (%)	
Overcorrection	8 (40%)	1 (5%)	0.008^††^
Undercorrection	0 (0%)	1 (5%)	0.31^†^
Surgical success	12 (60%)	18 (90%)	0.03^††^

^†^*P*-value > 0.05 is not significant, ^††^*P*-value < 0.001 is highly significant, ^*^Chi-squared/Fisher exact test

## Discussion

Congenital ptosis represents a special type of ptosis due to its unique characteristics. It occurs due to replacement of variable amounts of normal levator muscle fibers with fibro fatty tissue which adversely affects the action of the muscle [5]. Therefore, the choice of the surgical technique depends mainly on the levator action. For decades, levator resection has been considered the standard technique for cases with a levator function of at least 5 mm [6]. Despite its satisfactory outcomes, the classic procedure involves significant dissection with severing of the medial and lateral horns of the levator muscle. The current trend towards less invasive surgery has led to the evolution of minimal incision ptosis surgery. Small incision external levator resection is now widely used for aponeurotic ptosis with good success rates. However, only few studies have applied this technique in congenital ptosis, probably due to the high learning curve related to the narrow surgical field [7]. Levator muscle plication is another technique that requires minimal dissection and can be used for ptosis with a similar degree of levator function. It is nowadays favored by many surgeons for cases of congenital ptosis as it is simple, easier to learn and reversible [3].

The amount of ptosis correction is directly related to the amount of levator resection/plication. However, the exact amount of resection/plication needed to achieve the desired postoperative lid height is difficult to calculate. This is because the patient is usually a child, and it may be difficult to accurately assess the degree of ptosis and levator function [1]. Also, the operation is performed under general anesthesia that alters the tone of the ocular muscles making intraoperative adjustment less reliable [8].

In the past, numerous methods were introduced to estimate the suitable amount of levator resection. Berke [9]proposed a method based on the levator function, with best results achieved in patients with good or fair levator function. Carraway [10] suggested that for every 1 mm of ptosis, 4 mm of resection should be performed. A more recent study by Ho et al. [11] proposed that good surgical outcomes can be obtained considering only the preoperative MRD1. Unfortunately, none of these methods gained universal acceptance by oculoplastic surgeons. This is because no single method is able to achieve reproducible postoperative results. Furthermore, they target levator muscle resection, and till date, there has been no specific method especially developed for the levator plication technique.

In levator muscle plication, the medial and lateral horns of the levator muscle are kept intact. Also, the function of Muller’s muscle is preserved. Hence, in contradiction to classic levator muscle resection, this technique aims to strengthen the action of the levator muscle without alteration in the natural lid physiology [12]. We therefore believe that formulas used to calculate the amount of levator resection cannot achieve the same outcomes when used for levator plication. The rationale of this algorithm was based on previous postoperative results when applying the traditional formula. Upon reviewing our records, we found that most cases had a postoperative overcorrection of 1 mm in MRD1. Therefore, our hypothesis was to decrease the intraoperative amount of plication by 3–4 mm to avoid this postoperative overcorrection. When implementing this rationale, we found that patients requiring < 15 mm plication achieved best results by decreasing the plication by 3 mm (mostly those with good levator function) while patients requiring > 15 mm plication achieved best results by decreasing the plication by 4 mm (mostly those with fair levator function). This was the basis of the hypothesis upon which we performed our study. The aim of this study was to evaluate the outcomes of a novel algorithm we created for the levator muscle plication technique based on our hypothesis and to compare it with the formula we usually use for levator muscle resection.

In our study, the mean amount of levator muscle plication was 16.98 ± 2.44 mm in group A (traditional formula) and 13.48 ± 2.42 mm in group B (novel algorithm). This difference was highly statistically significant (*P* < 0.001). At the 1st postoperative week, the mean MRD 1 was 4.95 ± 0.37 mm and 4.08 ± 0.64 mm in group A and group B respectively. The mean MRD 1 at the 1st week was greater in group A than group B and this difference was highly significant (*P* < 0.001). This was due to overcorrection that occurred in 8 (40%) eyes in group A and 1 (5%) eye in group B. There was a statistically significant difference between the two groups as regards the incidence of overcorrection (*p* = 0.008). 12 (60%) eyes in group A versus 18 (90%) eyes in group B reached surgical success. The difference between the two groups was statistically significant (*p* = 0.03).

Our study revealed that the novel algorithm we created for levator muscle plication is effective with a high success rate (90%). Our results are consistent with recent studies that have reported surgical success with transcutaneous levator plication in congenital ptosis to range from 85–95% [2, 3, 12]. This new algorithm depends on the degree of ptosis and levator function, both of which are important factors that affect the results of ptosis surgery [8, 13].

We didn’t report any cases of recurrence till the last follow-up visit. The use of non-absorbable (Ethibond) suture to plicate the levator muscle gives long standing results and prevents recurrence [2]. All patients in our study had a good postoperative aesthetic appearance. We believe that the technique of levator plication creates a fold of the muscle that gives natural fullness to the eyelid and guards against a deep sulcus.

There are several limitations to our study, namely its retrospective nonrandomized nature and the relatively small sample size. Future prospective studies with larger sample sizes are needed to further validate our results. Also, the studied population was limited to patients with simple congenital ptosis to obtain reliable results. Therefore, we cannot be certain if this nomogram can be applicable to other types of ptosis.

## Conclusion

Our novel nomogram is effective in calculating the amount of levator muscle plication needed to achieve a satisfactory surgical outcome in cases with simple congenital ptosis.

## Data Availability

Data available on request from the corresponding author, Email: masalamah@zu.edu.eg. Phone: 00201148918518. ORCID: 0000-0003-2211-3383.
